# Acquired resistance to crizotinib in novel CDK14-ALK and CLTC-ALK fusions of ALK-positive large B-cell lymphoma identified by next-generation sequencing

**DOI:** 10.1080/15384047.2023.2271212

**Published:** 2023-10-31

**Authors:** Yuxue Xia, Lu Zhang, Wenjuan He, Huaxiong Pan, Jun Fang, Guohui Cui

**Affiliations:** a Institute of Hematology, Union Hospital, Tongji Medical College, Huazhong University of Science and Technology, Wuhan, China; b Department of Hematology, Dabieshan District Medical Center, Huanggang, China; c Department of Pathology, Union Hospital, Tongji Medical College, Huazhong University of Science and Technology, Wuhan, China

**Keywords:** ALK, large B-cell lymphoma, crizotinib, CLTC, CDK14, MFHAS1

## Abstract

Anaplastic lymphoma kinase-positive large B-cell lymphoma (ALK^+^ LBCL) is a rare subtype of non-Hodgkin lymphoma. ALK inhibitors are being tried to treat recurrent/refractory ALK^+^ LBCL. A majority of patients with ALK^+^ tumors respond to crizotinib, but partial cases ultimately develop resistance about a year later. Here, we report a case of ALK^+^ LBCL carrying a new fusion gene involving CDK14 and ALK, CLTC-ALK gene rearrangements and MTOR gene mutation. The patient had progressive disease after combination of crizotinib and chemotherapy treatment about 5.5 months later, accompanied by reduced abundance of CDK14-ALK, increased abundance of CLTC-ALK and a novel MFHAS1 gene mutation. However, MTOR mutation turned negative. The patient received alectinib combined with hyper-CVAD, then followed by alectinib as monotherapy for 21 months. The patient achieved partial response and remained in a stable condition. This case suggests that CDK14-ALK fusion gene may be more sensitive to crizotinib than CLTC-ALK fusion gene. MTOR is associated with the anti-tumor mechanism of ALK inhibitors. MFHAS1 gene mutation and/or CLTC-ALK gene copy number amplification may involve resistance to crizotinib. Furthermore, alectinib may inhibit the carcinogenicity of these gene changes and improve the prognosis of ALK^+^ LBCL.

## KEY POINTS

The novel CDK14-ALK fusion gene in ALK+ LBCL was sensitive to crizotinib.

MFHAS1 gene mutation and/or CLTC-ALK gene copy number amplification may involve resistance to crizotinib.

## Introduction

Anaplastic lymphoma kinase (ALK) is a transmembrane receptor type protein tyrosine kinase, which belongs to the superfamily of insulin receptors. Crizotinib is a first-generation inhibitor of ALK, ROS1 and c-Met. Second-generation inhibitor alectinib has been granted a breakthrough therapy designation by the FDA due to its effectiveness for most ALK mutations, stronger targeting ability and greater advantages in intracranial disease control.^1,2^ ALEX studies showed that median progression-free survival times were 34.8 months with alectinib and 10.9 months with crizotinib in untreated ALK^+^ NSCLC.^3^ Currently, there are no case reports of alectinib for treating ALK^+^ LBCL.

## Case description

**Figure 1.**
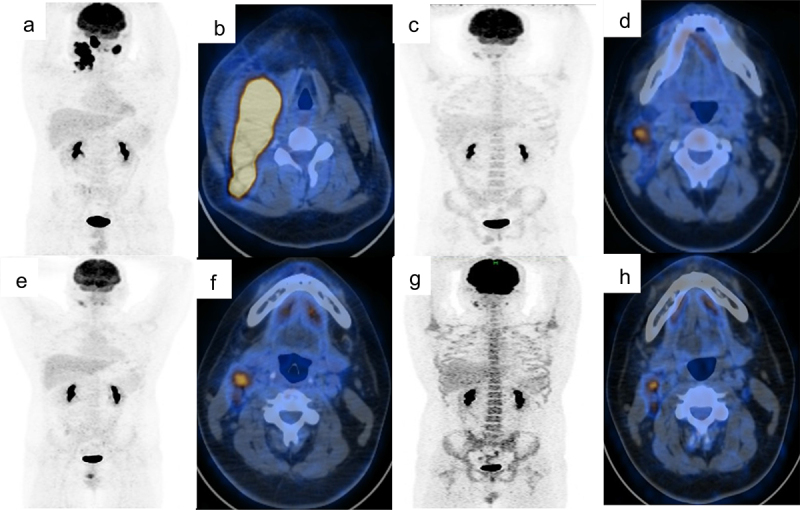
PET-CT test results before and after therapy.

A 15-year-old male patient sought care for gradual enlargement of bilateral cervical mass. The longest diameter of the right cervical mass was 13 cm with tenderness. There were no sweat, fever and weight loss. Past medical history was no special. Lactate dehydrogenase (LDH) level was 585 U/L. Enhanced CT scans revealed multiple bulky cervical lymph nodes with partial integration (4.6 cm × 4.4 cm). PET-CT displayed that multiple enlarged cervical lymph nodes were partial fusion with increased metabolic activity (Figure 1a,b).

a,b. Baseline assessment of PET-CT. There were right parapharyngeal and bilateral cervical lymph nodes enlargements with partial fusion into a mass (1.1 cm × 1.5 cm, SUVmax 5.0–6.2). c,d. Four courses of treatment later, the right parapharyngeal lymph nodes (1.8 cm × 1.2 cm) showed no abnormal increase in metabolism. The multiple lymph nodes in the II area of the right neck were smaller than the previous and the metabolic activity was lower than baseline, but still there was an abnormal increase (SUVmax 2.4–6.0). e,f. Six courses of treatment later, the multiple lymph nodes in the levels II area of the right neck had increased metabolic activity (1.6 cm × 1.3 cm, SUVmax 3.4–8.8). g,h. Eight courses of treatment later, the metabolic activity of the right parapharyngeal lymph nodes was even higher (1.8 × 1.3 cm, SUVmax 4.9) and the metabolic activity of the left cervical lymph nodes increased (0.6 × 0.8 cm, SUVmax 4.8), but the metabolic activity of the right cervical lymph nodes (1.2 × 1.0 cm, SUVmax 2.9-6.3) was inhibited than the last assessment.

Bone marrow cytology and EBER were negative. Biopsy of the right cervical lymph node was performed. The tumor cells were positive for ALK, CD138, EMA, MUM-1, OCT2, Bob.1, and CD38 (partial) by immunohistochemistry. The neoplastic cells were negative for CD30, CD20, PAX5, and CD5. Ki-67 proliferation index was 60%. Next-generation sequencing (NGS) detected CDK14-ALK (C6; A20, abundance was 20.16%) and CLTC-ALK (C31; A20, abundance was 2.71%) rearrangements and MFHAS1 (exon1:c.118_135dup, abundance was 0.32%) and MTOR (exon39:c.5490_5501del, abundance was 44.97%) gene mutation. Therefore, the patient was diagnosed with “ALK^+^ LBCL, Stage II, Group A”.

**Figure 2.**
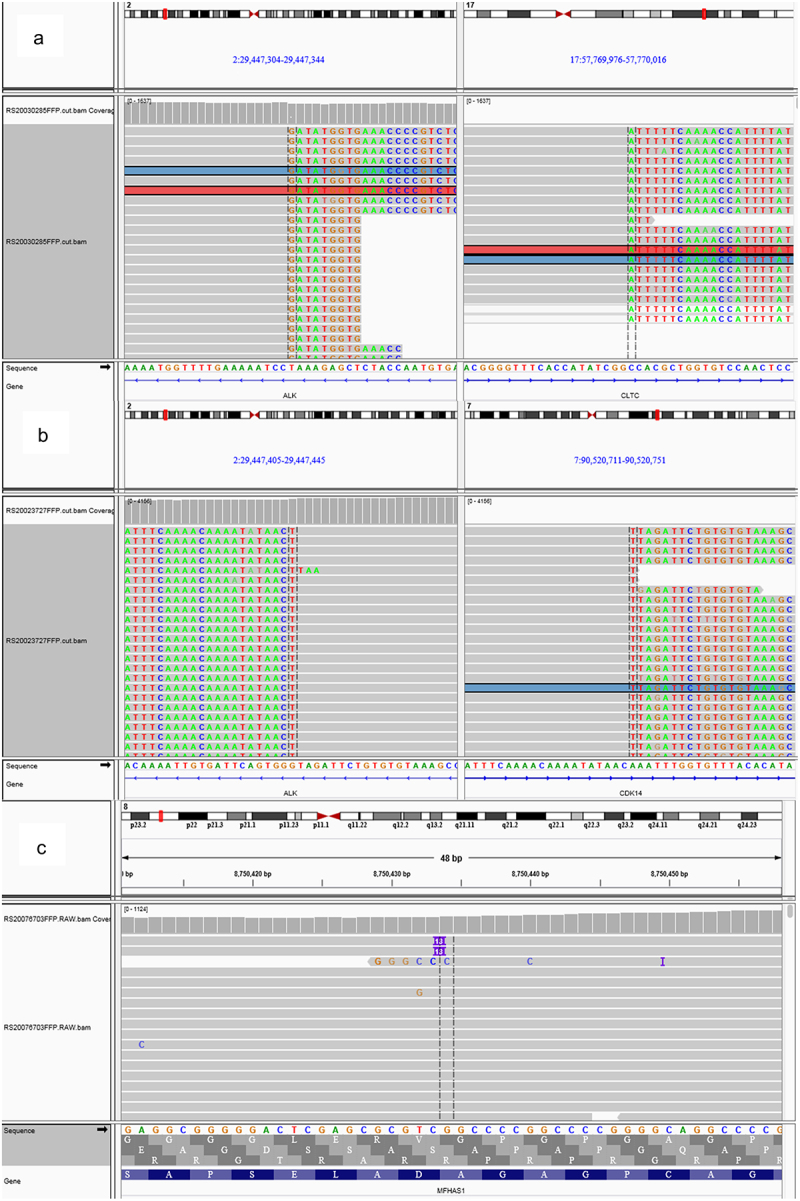
NGS. a. CLTC-ALK (C31; A20) rearrangement, b. CDK14-ALK (C6; A20) rearrangement, c. MFHAS1 (exon1:118_135dup) gene mutation.

In view of ALK rearrangement, the patient received CDOP regimen combined with crizotinib (250 mg, bid). After two cycles of treatment, the mass significantly reduced. Enhanced CT scanning suggested partial response (PR), and the sum of the products for the longest perpendicular diameters (SPD) had a 64.13% decrease from baseline. LDH level quickly dropped from 387 U/L to 265 U/L. Four courses later, PET-CT unveiled PR with decreased volume and metabolism of partial lymph nodes (Figure 1c,d). Then, the patient received two cycles of CHOPE combined with crizotinib. In the context of COVID-19, the patient could not carry out assessment, and only treated at home with crizotinib monotherapy. Two months later, PET-CT showed increased metabolism of the right parapharyngeal and cervical lymph nodes, which suggested progressive disease after 5.5 months of treatment (Figure 1e,f). Due to the impact of COVID-19 epidemic, tissue biopsy could not be carried out. Therefore, he was administrated DHAP regimen as second-line chemotherapy. After two cycles of DHAP, PET-CT result showed that the metabolism of the right parapharyngeal lymph nodes was even higher and the metabolism of the left cervical lymph nodes increased, but the metabolism of the right cervical lymph nodes (1.8 × 1.3 cm, SUVmax 2.9–6.3) decreased, which suggested refractory lymphoma (Deauville score 4–5, Figure 1g,h). Under effective control of COVID-19 epidemic, right cervical lymph node biopsy was performed immediately and B-cell-lymphoma-associated mutant genes at this site were detected by NGS. Immunohistochemistry showed that lymphoma cells were BCL2 positive, and other surface markers had no significant changes. Ki-67 proliferation index was 80%. NGS showed the abundance of CDK14-ALK decreased from 20.16% to 6.08%, CLTC-ALK increased from 2.71% to 6.34%, the abundance of MFHAS1 increased from 0.32% to 31.01%, and MTOR gene turned negative (Figure 2). Considering CLTC-ALK fusion gene copy number amplification, the patient stopped using crizotinib instead of alectinib. Alectinib (600 mg, bid) was administrated combined with one cycle of Hyper-CVAD regimen. Then, the patient and his families refused chemotherapy because of chemotherapy-related adverse reactions. Then, alectinib was sustainedly used as monotherapy. A month later, the patient’s LDH level dropped rapidly from 387 U/L to normal levels. Five months after the use of alectinib, the right parapharyngeal lymph nodes necrosis and calcification occurred. SPD had a 81.0% decrease from baseline by enhanced CT scanning. The patient is in stable condition during the 28 months of maintaining treatment.

## Result and discussion

In ALK^+^ LBCL, the most frequent ALK gene rearrangement was caused by t(2;17) (p23; q23), producing the CLTC-ALK rearrangement in 75% of cases.^4^ There are other fusion partners, such as NPM, SQSTM1, SEC31A, RANBP2 and IGL.^5^ CDK14-ALK fusion gene found in this case was novel and firstly reported. The specific carcinogenic mechanism is not clear. For this case, the abundance of CDK14-ALK decreased after crizotinib treatment, indicating that the fusion gene was sensitive to crizotinib. Overexpression of CDK14 is associated with cell proliferation and invasion in breast cancer.^6^ Genome-wide expression profile analysis showed that CDK14 gene was positively correlated with PD-L1 expression in DLBCL.^7^ Due to limited research on CDK14 in lymphoma, the pathogenesis of the gene needs further research.

The abundance of CLTC-ALK fusion gene increased after crizotinib resistance in our case. This fusion protein is constitutively active and has the ability to promote malignant transformation.^8^ Subsequently, ALK inhibitor was reported to induce apoptosis of CLTC-ALK-positive DLBCL cells and sustain tumor regression in the xenotransplant tumor model.^9^ However, the relationship between abundance changes of CLTC-ALK and resistance mechanism was not reported. In 1 of the 15 advanced ALK^+^ NSCLC cases examined, ALK FISH revealed high-level gene amplification. Besides, the team previously found that amplification of wild-type EML4-ALK causes resistance to crizotinib in NSCLC cells.^10^ In view of the above, the sensitivity of CLTC-ALK to crizotinib may be weaker than that of CDK14-ALK. The copy number amplification of CLTC-ALK fusion gene may be a risk factor of crizotinib resistance for this case, and alectinib was effective for these mutations.

MFHAS1 gene mutation has not been reported in ALK^+^ LBCL. Hiroyuki Tagawa^11^ found that MFHAS1 gene was involved in t(8;14)(p23.1;q21) in immunoblastic B-cell lymphoma cell line OCI-LY8. MFHAS1 was found to be a potential oncogene for primary mediastinal B cell lymphoma by whole exon sequencing in 14 patients, relapsed/refractory B-cell lymphoma patients.^12^ MFHAS1 refers to innate immunity by Toll-like receptor-dependent signaling^13^ and macrophage polarization by activating JNK and p38 pathways.^14^ Besides, MFHAS1 could induce M2 polarization of TAMs and subsequent STAT6 and KLF4 activation to promote CRC progress.^15^ In our case, the abundance of MFHAS1 gene mutations was very low before treatment (<2%), and increased to 31.01% after progression, indicating that MFHAS1 may associate with crizotinib resistance and lymphoma progression.

MTOR gene turned negative after crizotinib treatment for our case, suggesting that MTOR is associated with the mechanism of crizotinib inhibition of ALK-positive tumor cells. MTOR signaling pathways are reported to be associated with antitumor effects of ALK inhibitors. While crizotinib induces apoptosis in EML4-ALK-positive lung cancer cell lines, the expression levels of downstream signaling proteins of the MTOR signaling pathway, such as the activated forms of PI3K, AKT and mTOR, were inhibited.^16^ Meanwhile, the synergistic effect of ALK inhibitors and MTOR inhibitors was observed in internal and external studies of ALK lymphoma cell lines.^17^

Furthermore, this case clarifies the pivotal role of high-throughput sequencing in the discovery of novel gene mutation and the exploration of drug resistance mechanism in the era of precise medicine. We are trying to explore the resistance mechanism of this case to provide experience in the treatment of such lymphoma.

## Data Availability

The data that support the findings of this study are openly available in [repository name e.g “figshare”] at http://doi.org/[doi], reference number [reference number].
